# Evolution and roles of cytokinin genes in angiosperms 1: Do ancient *IPTs* play housekeeping while non-ancient *IPTs* play regulatory roles?

**DOI:** 10.1038/s41438-019-0211-x

**Published:** 2020-03-01

**Authors:** Xiaojing Wang, Shanshan Lin, Decai Liu, Lijun Gan, Richard McAvoy, Jing Ding, Yi Li

**Affiliations:** 1 State Key Laboratory of Crop Genetics and Germplasm Enhancement and the College of Horticulture, Nanjing Agricultural University, Nanjing, P. R. China; 2 College of Life Sciences, Nanjing Agricultural University, Nanjing, P. R. China; 3 Department of Plant Science and Landscape Architecture, University of Connecticut, Storrs, CT 06269 USA

**Keywords:** Evolution, Plant evolution

## Abstract

Isopentenyltransferase (IPT) genes, including those encoding *ATP/ADP-IPT*s and *tRNA-IPT*s, control the rate-limiting steps of the biosynthesis of *N*^6^-(Δ^2^-isopentenyl)adenine (iP)-type and *trans*-zeatin (*t*Z)-type cytokinins and *cis*-zeatin (*c*Z)-type cytokinins, respectively. However, the evolution and roles of these *IPT*s in angiosperms are not well understood. Here, we report comprehensive analyses of the origins, evolution, expression patterns, and possible roles of *ATP/ADP-IPTs* and *tRNA-IPT*s in angiosperms. We found that Class I and II *tRNA-IPT*s likely coexisted in the last common ancestor of eukaryotes, while *ATP/ADP-IPT*s likely originated from a Class II *tRNA-IPT* before the divergence of angiosperms. *tRNA-IPT*s are conservatively retained as 2–3 copies, but *ATP/ADP-IPT*s exhibit considerable expansion and diversification. Additionally, *tRNA-IPT*s are constitutively expressed throughout the plant, whereas the expression of *ATP/ADP*-*IPT*s is tissue-specific and rapidly downregulated by abiotic stresses. Furthermore, previous studies and our present study indicate that *ATP/ADP*-*IPT*s and their products, iPs/*t*Zs, may regulate responses to environmental stresses and organ development in angiosperms. We therefore hypothesize that *tRNA-IPT*s and the associated *c*Zs play a housekeeping role, whereas *ATP/ADP-IPT*s and the associated iP/*t*Z-type cytokinins play regulatory roles in organ development and stress responses in angiosperms, which echoes the conclusions and hypothesis presented in the accompanying study by Wang, X. et al Evolution and roles of cytokinin genes in angiosperms 2: Do ancient CKXs play housekeeping roles while non-ancient CKXs play regulatory roles? *Hortic Res* 10.1038/s41438-020-0246-z.

## Introduction

Cytokinins (CKs) are a class of plant hormones that play essential roles in many aspects of plant development, including the delay of leaf senescence^1^, root proliferation^2,3^, apical dominance^4^, shoot meristem function^5–7^, regulation of reproductive meristem activity^8^, fruit development^9^, and nutritional signaling^10,11^. In addition, CKs play important and complex roles in environmental stress responses^12,13^. There are two major types of naturally occurring CKs, the *cis*-zeatin (*c*Z) type and the iP [*N*^6^-(iP]/*trans*-zeatin (*t*Z) type^14^. In many plants, iPs and *t*Zs are the most prevalent CKs in most tissues and stages of the lifespan, while *c*Z-type CKs are present only in minor quantities^15^. Recent studies have demonstrated that *c*Zs are the predominant CKs in some plants, such as rice and maize, or in certain developmental stages associated with limited growth^16^. The presence of iP-type and *t*Z-type CKs can vary greatly between tissues, developmental stages, and environmental conditions^17–19^.

Isopentenyltransferases (IPTs) catalyze the first and rate-limiting step of CK biosynthesis. IPTs can be classified into the adenylate (ATP/ADP-IPTs and AMP-IPTs) and tRNA types. The IPTs that use ATP, ADP, or AMP as their preferred substrates produce iP-type and *t*Z-type CKs, while the tRNA-type IPTs, which transfer isopentenyl groups to the *N*^6^ atom of adenines in tRNAs, produce *c*Z-type CKs^14,19^. In *Arabidopsis*, nine *IPT* genes have been identified (*AtIPT1–9*). Among them, *AtIPT1* and *AtIPT3*–*AtIPT8* encode ATP/ADP-IPTs, and the other two *AtIPT* genes, *AtIPT2* and *AtIPT9*, encode tRNA-IPTs. Mutations in two tRNA-type *IPT* genes, *AtIPT2* and *AtIPT9*, particularly in combination, result in plant chlorosis and a significant reduction in *c*Z-type CKs but do not affect the concentrations of iPs and *t*Zs^14,20^. By comparison, plants with mutant *ATP/ADP-IPT*s possess scarce amounts of iP-type and *t*Z-type CKs but contain slightly elevated levels of *c*Zs^14,21,22^. These mutant plants exhibit increased lateral root primordia and density, fewer leaves, reduced inflorescence, and decreased number of vascular bundles and are unable to form cambium^14,21,22^.

Genetically mutant plants with altered endogenous CK content display changes in plant tolerance to environmental stresses. A quadruple *ATP/ADP-IPT Arabidopsis* mutant *atipt1;3;5;7* with deficient levels of iPs and *t*Zs but slightly increased levels of *c*Zs displayed significantly enhanced salt and drought tolerance compared to that of wild-type plants^23^. Plants overexpressing CK degradation genes (CK oxidase/dehydrogenase genes, *CKX*s), which contain substantially reduced levels of iPs/*t*Zs and moderately reduced levels of *c*Zs, also show elevated tolerance to drought, salt, or heat stress^23,24^. These studies show that iP and *t*Z CKs are negative regulators of plant adaptation to environmental stresses. However, it has also been reported that if a stress-associated or senescence-associated promoter is used to drive the expression of an *AMP-IPT* or *ATP/ADP-IPT* gene, improved plant tolerance to drought, heat, and other stresses is observed^13^.

As previously reported^19,25^, *tRNA-IPT*s have been found in all major clades of bacteria and eukaryotes but not in archaea. In contrast, adenylate *IPT*s show a fragmented distribution. The functionally confirmed *ATP/ADP-IPT*s are found exclusively in flowering plants^14,26^. Based on the tree topology and taxonomic composition of the *IPT* gene family, Lindner et al.^25^ have suggested that eukaryotic *IPT*s most likely can be traced back to an ancestral α-proteobacterium that was involved in the initial endosymbiotic event that led to extant mitochondria. The ancestral eukaryotic *IPT* gene was duplicated, which resulted in two classes of *tRNA-IPT*s (Class I and II). The Class II tRNA-IPTs subsequently lost the capability to bind tRNAs and diversified into the extant ADP/ATP-IPTs found in flowering plants. Recently, Nishii et al.^27^ performed a broader sampling of *IPT* genes and hypothesized that Class I *tRNA-IPT*s represent the direct successors of bacterial *miaA* genes, while Class II *tRNA-IPT*s are derived from eukaryotic genes.

In this study, we comprehensively analyzed the differences between *ATP/ADP-IPT*s and *tRNA-IPT*s, including their taxonomic distribution, origin, evolutionary history, duplication mechanism, gene/protein structure, and expression patterns in tissues/organs and under various environmental stresses. Our results strongly suggest that *tRNA-IPT*s in angiosperms can be traced back to the initial endosymbiotic event, while *ATP/ADP-IPT*s are derived from *tRNA-IPT*s. In flowering plants, *tRNA-IPT*s are conservatively retained and constitutively expressed in all tissues and under various environmental conditions. On the other hand, *ATP/ADP-IPT*s have extensively expanded and functionally diverged, and their expression is tissue-/organ-specific and environmental stress-responsive. Our findings plus previously published results demonstrate that in angiosperms, *tRNA-IPT* genes and the associated *c*Z-type CKs most likely play housekeeping roles, while *ATP/ADP-IPT* genes and the associated iP/*t*Z-type CKs may be involved in regulating organ development and responses to environmental stresses.

## Results

### Genome-wide identification of *IPT* genes among the three domains of life

*IPT* genes have been identified in various groups of bacteria and eukaryotes but not in archaea^19^. To verify the absence of *IPT* homologs in archaea, we used TBLASTN to identify the IPPT domain in archaeal nucleotide databases. Putative IPPT domains were found in two archaeal species, *miscellaneous Crenarchaeota group archaeon SMTZ-80* and *candidate division MSBL1 archaeon SCGC-AAA382N08*, indicating that *IPT* homologs are also present in archaea. The former archaeal species containing an *IPT* homolog belongs to the TACK superphylum, which has recently been proposed to be the origin of eukaryotes, and the latter is a member of the unclassified euryarchaeota.

We next performed a broad and balanced sampling of a total of 59 representative species from major lineages of all three domains of life (eukaryotes, bacteria, and archaea, Table S1). *IPT* homologs in these species were identified. We found that the number of *IPT* homologs varies considerably among the species in Plantae, ranging from one copy in *C. reinhardtii* (green algae) and *C. merolae* (red algae) to nine copies in *Arabidopsis thaliana* (eudicot) and 11 copies in *Z. maize* (monocot). By comparison, the number of *IPT* homologs in the other organisms, i.e., nonplant eukaryotes, bacteria, and archaea, does not vary greatly and ranges from 0 to 3 copies (Table S1). More than 90% (20/22) of the bacteria and nonplant eukaryotes sampled contain putative *IPT* gene(s) (mostly a single copy), while none of the *IPT* homologs could be found in the sampled archaea except the two identified by TBLASTN. Taken together, our results show that *IPT* homologs are distributed sporadically in archaea but widely throughout bacteria and eukaryotes, and show significant expansions in land plants.

### Differences in the origin and distribution of *tRNA-IPT*s and *ATP/ADP-IPT*s

**Fig. 1:**
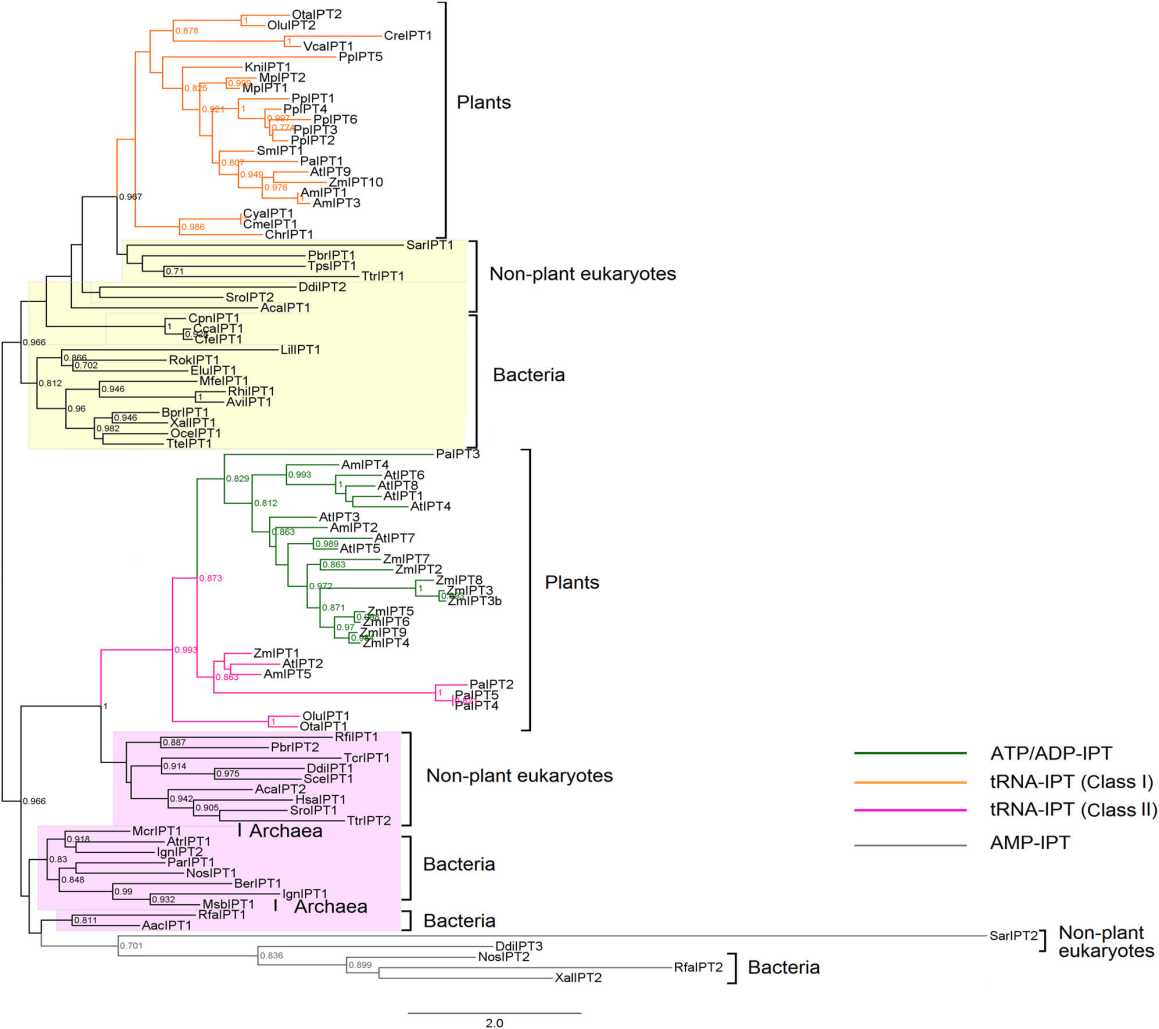
Phylogeny of the *IPT* gene family in all three domains of life. Phylogenetic tree of *IPT* genes from major lineages of bacteria, archaea, and eukaryotes constructed in PhyML. Support values (Shimodaira–Hasegawalike test) >0.5 are indicated at the nodes. Color coding: green lines, *ATP/ADP-IPT*; orange lines, Class I *tRNA-IPT*; pink lines, Class II *tRNA-IPT*; gray lines, *AMP-IPT*; orange and pink backgrounds, putative Class I and II *tRNA-IPT* homologs, respectively

To explore the evolutionary history of the *IPT* genes, an unrooted ML phylogeny comprised of IPT homologs from 59 representative species was constructed using PhyML (Fig. 1). Based on *IPT*s with known functions, the entire *IPT* gene family could be divided into three subfamilies, designated *tRNA-IPT*, *ADT/ATP-IPT*, and *AMP-IPT*^19^. According to our phylogeny, the *AMP-IPT* subfamily includes only members of *D. discoideum* and several bacteria, including *Agrobacteria*, and the *ATP/ADP-IPT* subfamily is confined to seed plants (Table 1). In comparison, the *tRNA-IPT* subfamily has a much wider taxonomic distribution.

**Table 1:** Phyletic distribution of *ATP/ADP-IPT*, *AMP-IPT*, and Class I and Class II *tRNA*-*IPT* homologs

Domain	Supergroup	Lineage	*ATP/ADP -IPT*	*tRNA-IPT*		*AMP -IPT*
				I	II	
Eukaryota	Plantae	Angiosperms	+	+	+	−
		Gymnosperms	+	+	+	−
		Lycophytes	−	+	−	−
		Liverworts	−	+	−	−
		Mosses	−	+	−	−
		Green algae	−	+	+	−
		Red algae	−	+	−	−
	SAR		−	+	+	−
	Excavata		−	−	+	−
	Amoebozoa		−	+	+	−
	Opisthokonta		−	+	+	−
	Incertae sedis		−	+	+	−
Bacteria			−	+	+	+
Archaea			−	−	+	−

*Note*: Plus (+) and minus (–) indicate whether the gene was detected in the corresponding lineage/supergroup/domain

The two classes of *tRNA*-*IPT* genes (referred to as Class I and II hereafter) categorized by Lindner et al.^25^ are located in two clades and include members of various species (Fig. 1). The Class I *tRNA-IPT* homologs can be found in all supergroups of eukaryotes except Excavata (Table 1). In particular, every species containing plastids has at least one Class I *tRNA-IPT* (Table S1), revealing the universal distribution of this type of *IPT* gene in photosynthetic organisms. Within the phylogeny, all eukaryotic Class I *tRNA-IPT*s form a clade that branches with the *tRNA-IPT* homologs from bacterial species belonging to proteobacteria and four other phyla (Fig. 1 and Table S1). It is worth noting that these five bacterial phyla have relatively close relationships in the recent tree of life^28^. Therefore, the bacterial homologs of Class I *tRNA-IPT*s may be traced back to the ancestor of these proteobacteria-related bacterial species.

In contrast, the Class II *tRNA-IPT* subfamily includes homologs from seed plants and two prasinophyte algae in Plantae and all nonplant eukaryotic supergroups (Table 1). In particular, the Class II *tRNA-IPTs* and *ATP/ADP-IPT*s in seed plants form a clade that is a sister to that of the putative Class II *tRNA-IPT*s in prasinophyte algae and nonplant eukaryotes. This phylogenetic relationship indicates that these two types of *IPT* genes in seed plants have a common ancestor, and the *ATP/ADP-IPT*s are likely derived from the Class II *tRNA-IPT*s. Additionally, the eukaryotic Class II *tRNA-IPT* clade branches with a cluster of *IPT*s, including *AMP-IPT*s and bacterial and archaeal *tRNA-IPT*s. Hence, we categorized these bacterial and archaeal *tRNA-IPT*s as putative Class II members. The bacterial species containing these putative Class II *tRNA-IPT*s include cyanobacteria, actinobacteria, and other species except proteobacteria and those species that are phylogenetically closely related to proteobacteria.

In other words, all the bacteria sampled contain only one type of *tRNA-IPT* homolog, and those with the same type of *tRNA-IPT* homolog are phylogenetically more closely related than those without a homolog. In the sampled bacteria, there are four bacterial species with two *IPT* copies, all of which have one *AMP-IPT* homolog and one *tRNA-IPT* homolog of either of the two classes. In contrast, all the nonplant eukaryotes with more than one *IPT* copy contain both types of *tRNA-IPT*s, with only one exception. Moreover, these nonplant eukaryotic species belong to different supergroups that diverged at the base of the eukaryote phylogenetic tree, indicating that the coexistence of the two classes of *tRNA-IPT*s probably originated before the divergence of these supergroups.

### Differences in evolutionary patterns between *tRNA-IPT*s and *ATP/ADP-IPT*s in land plants

**Fig. 2:**
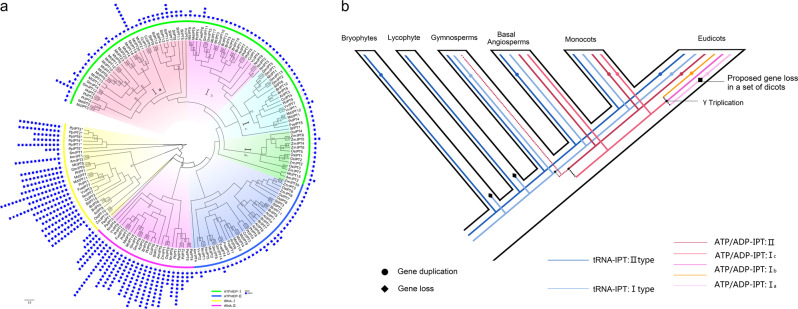
Phylogeny and inferred expansion history of the *IPT* gene family. **a** The unrooted ML phylogenetic tree constructed by using PhyML based on the full-length amino acid sequences of the IPT proteins from 19 representative land plants. Bootstrap values >70 are shown. Color coding for the different groups is indicated at the bottom. **b** Inferred expansion history of the *IPT* gene family in land plants. Inferred duplications and proposed losses are shown with solid circles and black diamonds, respectively. Duplication events that occurred before the divergence of different lineages are marked by black filled circles, whereas lineage-specific duplication events are marked by circles filled with the lineage-specific colors. Colored lines represent the respective subgroups in Table S2. Dotted lines indicate that the generation of *ATP/ADP-IPTs* in gymnosperms is undetermined

Our identification shows that the *IPT* genes have undergone considerable expansions, specifically in land plants. To further investigate the evolution of *IPT* genes in land plants, we identified 171 *IPT*s from 19 sequenced species belonging to every major lineage of land plants (Table S2) and constructed an unrooted ML tree using PhyML (Fig. 2a). According to the tree topology and that of the known *IPT*s, the land plant *IPT* gene family was classified into four groups: *tRNA-IPT* I, *tRNA-IPT* II, *ATP/ADP-IPT* I, and *ATP/ADP-IPT* II. The members of the *tRNA-IPT* I group are distributed throughout all the main lineages of land plants, while those of the *tRNA-IPT* II group are confined to seed plants, which is consistent with the distribution of the two *tRNA-IPT* classes in the phylogeny shown in Fig. 1. The total number of *tRNA-IPT* genes in each flowering plant species is either two or three (Table 1), revealing that a conserved number of this type of *IPT* gene is retained in angiosperm genomes. However, six and five *tRNA-IPT* genes were found in *Physcomitrella patens* (moss) and *Picea abies* (gymnosperm), respectively, indicating that lineage-specific expansions have occurred in these two species/lineages.

The number of *ATP/ADP-IPT* genes varies from two copies in *Amborella trichopoda* to 21 copies in *Medicago truncatula*, which accounts for 40% of *IPTs* in the former and 91.3% of *IPTs* in the latter (Table 2). The high and increased percentages of *ATP/ADP-IPT*s indicate that the increased number of *IPT* genes in flowering plants is mainly due to the expansion of *ATP/ADP-IPT*s. According to the phylogeny, the two groups *ATP/ADP-IPT* I and II contain each of the two *ATP/ADP-IPT* genes in the basal angiosperm *A. trichopoda* (Fig. 2a), respectively, suggesting that two ancestral *ATP/ADP-IPT*s were likely present in the last common ancestor of angiosperms. In addition, the *ATP/ADP-IPT* I group can be further divided into four subgroups, I_a_, I_b_, I_c_, and I_d_. The *ATP/ADP-IPT* I_a_–I_c_ subgroups contain only eudicot members, while the *ATP/ADP-IPT* I_d_ subgroup contains exclusively monocots, reflecting the lineage-specific duplications of angiosperm *ATP/ADP-IPT*s (Fig. 2b and Table S3). Notably, *PaIPT3*, the *P. abies* gene that is located in the *ATP/ADP-IPT* clade of the phylogeny shown in Fig. 1, branches with the *tRNA-IPT* II group in the land plant trees, which is supported by the low bootstrap value (Fig. 2a). Therefore, whether gymnosperms contain *ATP/ADP-IPT* homologs could not be determined from the phylogenetic analyses. Nevertheless, the existence and expansion of *ATP/ADP-IPT*s in flowering plants could be determined.

**Table 2:** Number of *tRNA-IPT*s and *ATP/ADP-IPT*s resulting from different duplication mechanisms in flowering plants

Species	*IPT*s	*tRNA-IPT*s (ancient)	*ATP/ADP-IPT*s (non-ancient)					
			Total	WGD	Tandem	Proximal	Dispersed	Singleton
*Medicago truncatula* (Mt)	23	2	21	2	8	5	6	0
*Glycine max* (Gm)	14	3	11	11	0	0	0	0
*Prunus persica* (Pr)	7	2	5	2	0	0	3	0
*Malusx domestica* (Md)	12	3	9	6	1	0	2	0
*Fragaria vesca* (Fve)	7	2	5	2	0	0	3	0
*Populus trichocarpa* (Pt)	9	2	7	4	0	0	3	0
*Citrus clementina* (Cc)	7	2	5	2	0	0	3	0
*Brassica rapa* (Br)	13	3	10	10	0	0	0	0
*Arabidopsis thaliana* (At)	9	2	7	2	0	0	5	0
*Vitis vinifera* (Vv)	7	2	5	2	0	0	3	0
*Solanum lycopersicum* (Sl)	15	2	13	2	6	2	3	0
*Nelumbo nucifera* (Nn)	8	3	5	4	1	0	0	0
*Zea mays* (Zm)	11	2	9	5	0	0	3	1
*Oryza sativa* (Os)	10	2	8	4	0	0	4	0
*Amborella trichopoda* (Am)	5	3	2	0	0	0	2	0
Total	157	35	122	58	16	7	40	1

We further explored the duplication mechanisms responsible for the expansion of *ATP/ADP-IPT* genes in the 15 angiosperms using the MCScanX package^29^. Each *ATP/ADP-IPT* gene was assigned to one of the five modes, including WGD/segmental duplication, tandem duplication, proximal duplication, dispersed duplication, and singleton (Table 2). Approximately 47.5% of the *ATP/ADP-IPT* genes from every dicot or monocot were shown to be involved in WGD gene duplications, demonstrating that WGD/segmental duplication is a primary mechanism. In contrast, tandem and proximal duplications accounted for 13.2% and 5.7% of the *ATP/ADP-IPT* duplications, respectively. In particular, 21 of the 23 tandem/proximal duplications, which belong to the *ATP/ADP-IPT* I_a_ subgroup, are derived from only two species, *M. truncatula* and *S. lycopersicum*. These results indicate that WGD/segmental duplication has extensively contributed to the expansion of *ATP/ADP-IPT* genes, while tandem duplication has given rise to lineage-specific expansions of the *ATP/ADP-IPT* genes in flowering plants.

### Differences in the gene and protein structures of *tRNA-IPT*s and *ATP/ADP-IPT*s

We next compared the structural differences between *tRNA-IPT*s and *ATP/ADP-IPT*s, beginning with the investigation of the exon–intron organization of *IPT* genes in 19 land plants (Fig. 2a). It is interesting to note that 77.7% of the 121 *ATP/ADP-IPT* genes contain no introns, and 85.2% of the remaining *ATP/ADP-IPT*s contain only a single intron. By comparison, 90% of the 50 *tRNA-IPT* genes have 6–8 introns. These results suggest that *ATP/ADP-IPT* genes are probably derived from an intron-free ancestral *tRNA-IPT* gene.

**Fig. 3:**
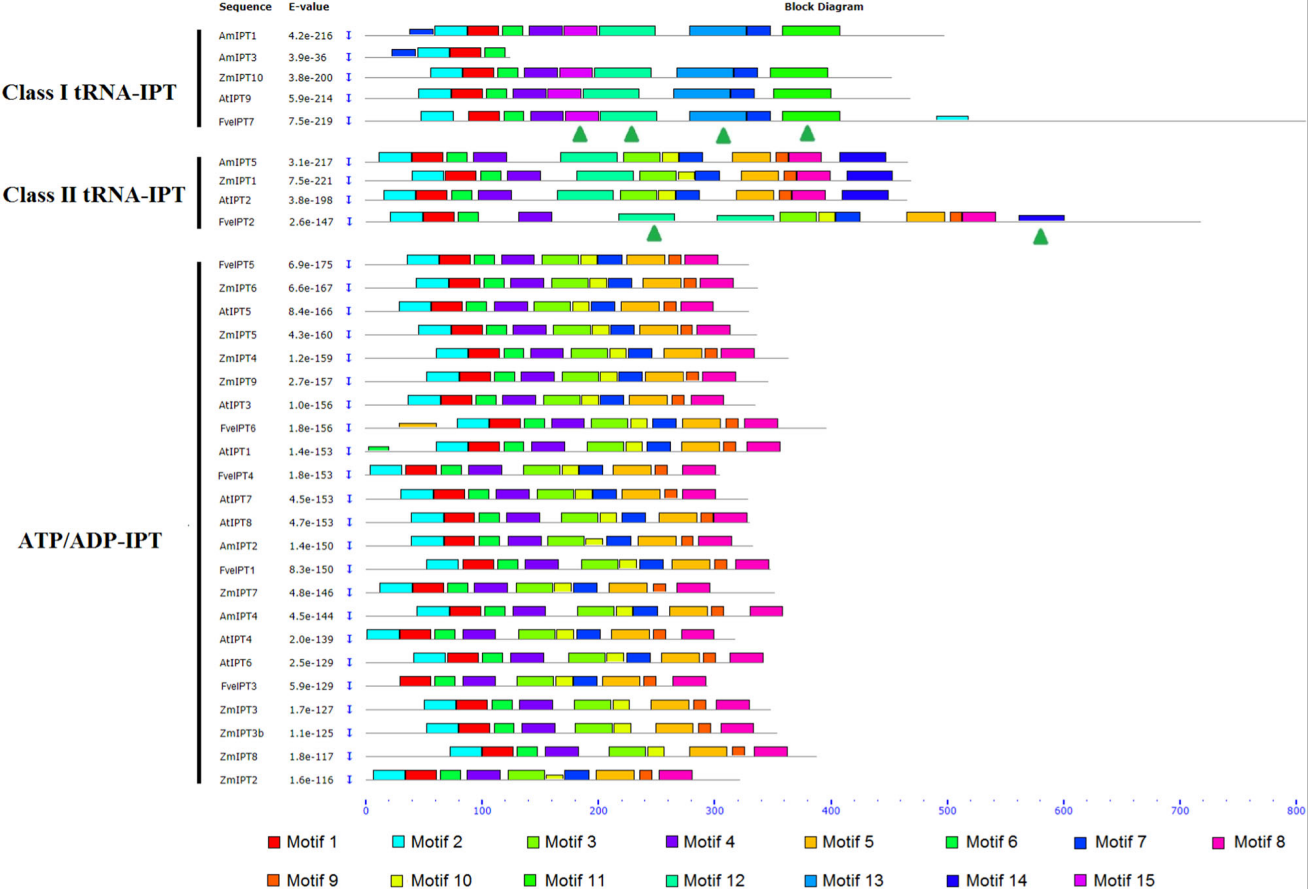
Schematic diagram of the amino acid motifs of IPT proteins from four representative flowering plants. Motif analysis was performed using MEME5.0.1 as described in the “Materials and methods” section. Color-filled rectangles show the individual predicted motifs, whose heights represent the levels of conservation among the motifs. Green solid triangles indicate the motifs specific to different groups

We further explored the motif composition of IPT proteins from four representative flowering plants (Figs. 3 and S1). Fifteen conserved motifs were identified. All three types of IPTs, Class I and Class II tRNA-IPTs and ATP/ADP-IPTs, show conserved motif structures among the members of every type. Motifs 11, 13, and 15 and motif 14 could be specifically detected in the Class I and Class II tRNA-IPTs, respectively, while no particular motif could be identified in the ATP/ADP-IPTs. The Class II tRNA-IPT proteins demonstrate a similar motif composition to the ATP/ADP-IPTs rather than the Class I tRNA-IPTs. In fact, the consensus motif structure of the ATP/ADP-IPTs and the Class II tRNA-IPTs is the same, except for motifs 12 and 14, providing direct evidence to support our above-described hypothesis that *ATP/ADP-IPT* genes are derived from the Class II *tRNA-IPT*s.

### Differences in the tissue/organ expression patterns of *tRNA-IPT*s and *ATP/ADP-IPT*s

**Fig. 4:**
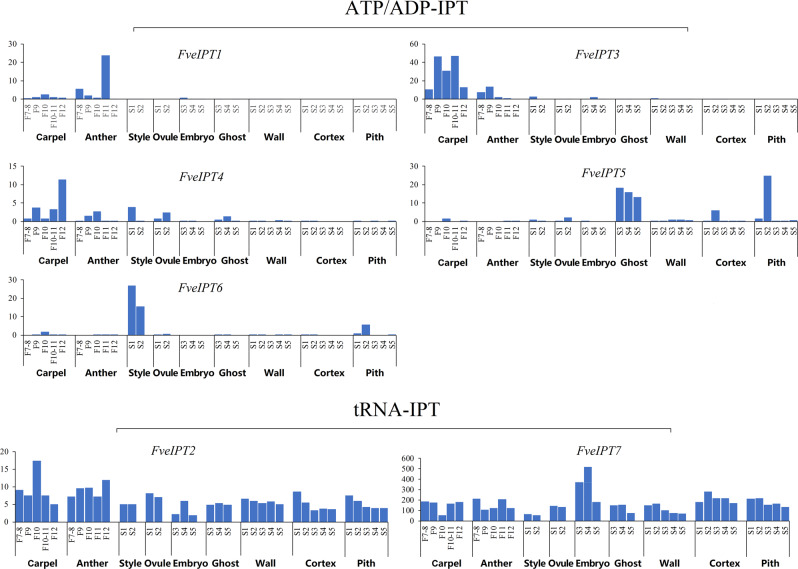
Expression profiles of *FveIPT* genes in different stages of *F. vesca* flowers and early-stage fruits. The *x*-axis indicates the different stages of *F. vesca* flowers and early-stage fruits, and the *y*-axis represents the mRNA length in kilobases per million mapped reads. Data were retrieved from the SGR database (http://bioinformatics.towson.edu/strawberry/). Expression levels were calculated according to the log2 scale. For a detailed description of the stages, please see http://bioinformatics.towson.edu/strawberry/newpage/Tissue_Description.aspx

To investigate the differences in the expression patterns of *tRNA-IPT*s and *ATP/ADP-IPT*s, we analyzed RNA-seq data from the basal angiosperm *A. trichopoda* and the eudicot woodland strawberry. In *A. trichopoda*, *AmIPT2* and *AmIPT4* belong to the *ATP/ADP-IPT* subfamily. The mRNA level of *AmIPT4* was low in vegetative organs and meristems and undetectable in female buds, while *AmIPT2* was highly expressed in vegetative organs and weakly expressed in meristems and female buds (Fig. S2). In *Fragaria vesca*, five genes (*FveIPT1, FveIPT3–6*) belong to the *ATP/ADP-IPT* subfamily. *FveIPT1, FveIPT3*, and *FveIPT4* were highly expressed in the various stages in carpel and anther. *FveIPT5* was highly expressed in ghost and in some stages of cortex and pith, and the mRNA level of *FveIPT6* was high in style and some stages of pith (Fig. 4). These different expression patterns indicate that functional diversification of the *ATP/ADP-IPT* genes after their expansion occurred in eudicots. By comparison, the three genes (*AmIPT1, AmIPT3*, and *AmIPT5*) in *A. trichopoda* and two genes (*FveIPT2* and *FveIPT7*) in *F. vesca*, which belong to the *tRNA-IPT* gene family, were relatively highly and stably expressed in all tissues, suggesting that *tRNA-IPT*s have a constitutive expression pattern similar to that of housekeeping genes.

**Fig. 5:**
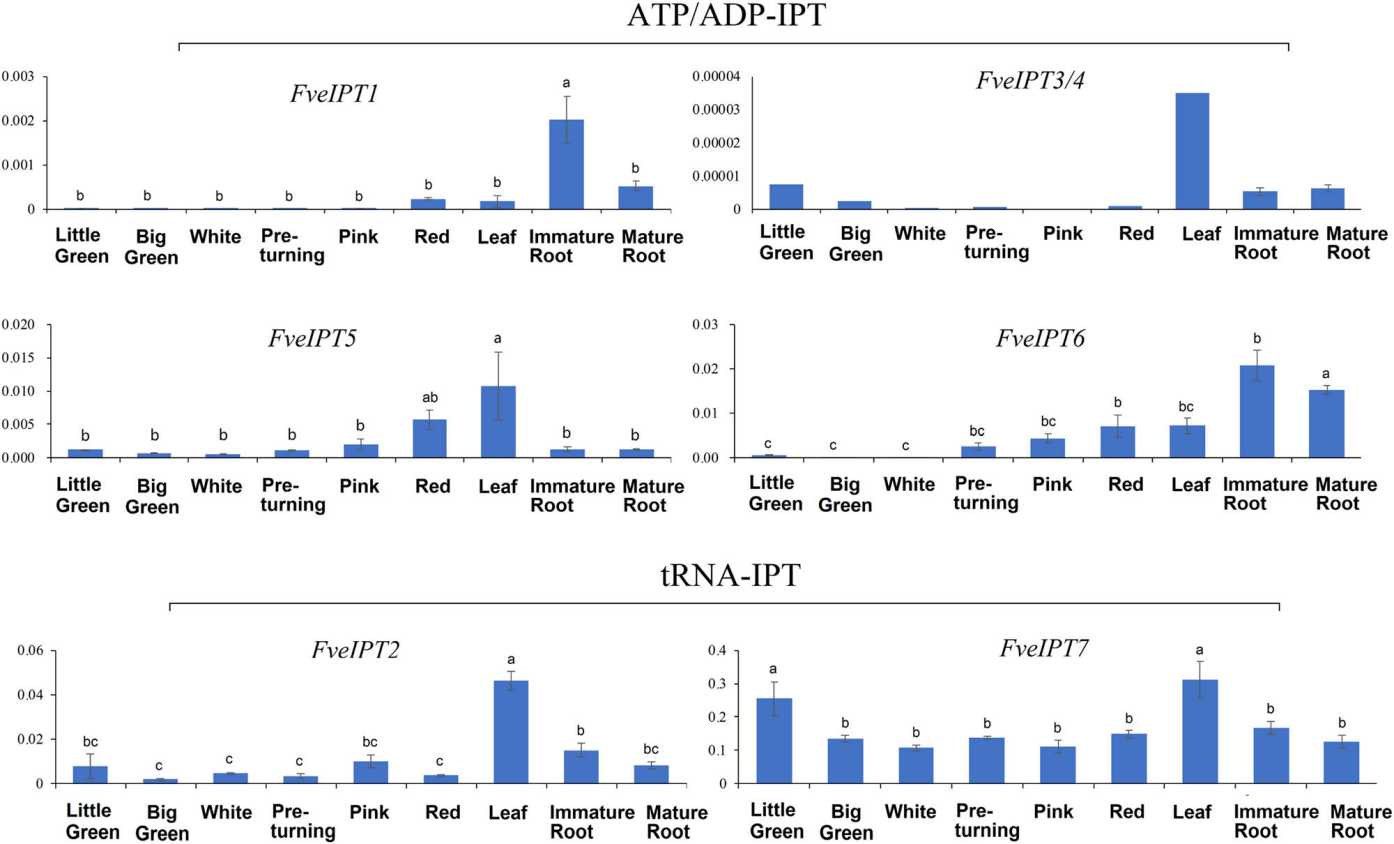
Expression profiles of the *FveIPT* genes in roots, leaves, and different early-stage fruits. Transcript expression was normalized to the expression of the *GAPDH* gene. The mean ± s.d. of three biological replicates is presented. The different lowercase letters above the bars indicate significant differences (*α* = 0.05, LSD) among the different tissues. Three biological replicates and three technical replicates were performed for each data point

We further used qRT-PCR to compare the differences in the expression patterns of the *F. vesca* *ATP/ADP-IPT* and *tRNA-IPT* genes in six stages of fruits (little green, big green, white, preturning, pink, and red) and three vegetative organs (leaves, immature roots, and mature roots, Fig. 5). Similar to the results of the above transcriptomic analyses, *ATP/ADP-IPT* genes showed quite diversified expression patterns. Although the transcript levels of *FveIPT1*, *FveIPT5*, and *FveIPT6* gradually increased during fruit ripening, *FveIPT1* exhibited higher expression levels in leaves, and *FveIPT5* and *FveIPT6* were highly expressed in immature roots. The expression of *FveIPT3/4* was undetectable in all these samples (<5 × 10^−5^). Meanwhile, the *tRNA-IPT* genes were constitutively expressed in different developmental stages of fruits, leaves, and roots. We further compared the variation amplitude of *FveIPT* gene expression in different organs. The variation amplitude of the expression of the *tRNA-IPT* genes (~2-fold) was substantially smaller than that of the *ATP/ADP-IPT* genes (30- to 130-fold). Therefore, the qPCR results showed that the *ATP/ADP-IPT* genes have quite diversified expression patterns, while the expression of the *tRNA-IPT* genes is relatively consistently high throughout the plant.

### Differences in the environmental stress responses of *tRNA-IPT*s and *ATP/ADP-IPT*s

**Fig. 6:**
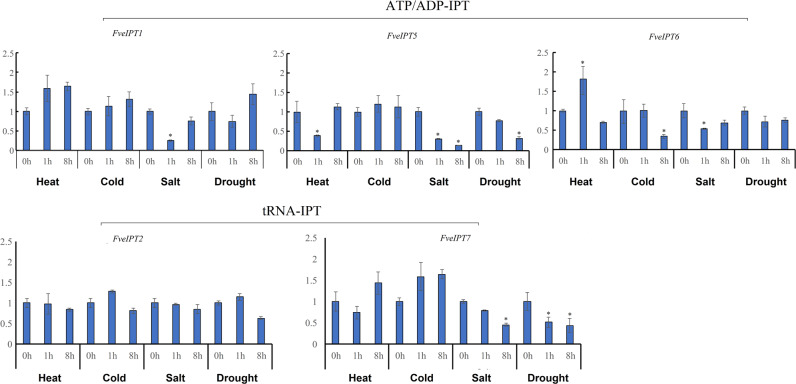
Expression profiles of *FveIPT* genes in response to environmental stresses. The expression levels relative to that of *GAPDH* were measured by qRT-PCR. Asterisks indicate that the corresponding gene was significantly upregulated or downregulated under the given condition (*p ≤ 0.05). Three biological replicates and three technical replicates were performed for each data point

CKs produced by IPT enzymes also play important roles in the regulation of plant responses to environmental stresses^30^. We further investigated the expression profiles of the *ATP/ADP-IPT* and *tRNA-IPT* genes under salt, dehydration, hot, and cold stress conditions (Fig. 6). Among the *ATP/ADP-IPT* genes, the expression of *FveIPT1* was significantly decreased (>4-fold and *P* < 0.001) after salt stress, and the expression of *FveIPT5* was significantly decreased after salt (>7-fold) and drought stress at 8 h (>3-fold). The transcription of *FveIPT6* was significantly decreased by more than 2–3-fold after salt, dehydration, and cold stress and was increased by 2-fold under heat stress. *FveIPT3/4* was undetectable after all four types of stress (<4 × 10^−5^). For the *tRNA-IPT* genes, the transcription of *FveIPT7* was reduced by ~2-fold after salt and dehydration stress (Fig. 6). The changes in *ATP/ADP-IPT* gene expression levels were drastic in response to environmental stresses, but much smaller changes were observed in the expression levels of *tRNA-IPT* genes under the same stress conditions. These results show that *ATP/ADP-IPT* genes may be involved in plant responses to environmental stresses, whereas *tRNA-IPT*s are minimally involved in stress responses.

## Discussion

IPT enzymes catalyze the first step of CK biosynthesis^26^. ATP/ADP-IPTs and tRNA-IPTs use different substrates and produce distinct biologically active CKs in flowering plants^31^. In the present study, we performed comprehensive analyses of the differences between these two types of *IPT* genes in terms of their taxonomic distribution, copy number variation, evolutionary history, duplication mechanism, tissue/organ expression, and environmental stress responses (Table 3). Based on the results here and those previously published by others, we hypothesize that *ATP/ADP-IPT*s are more important in regulating organ development and environmental stress responses, while *tRNA-IPT*s mainly play a housekeeping role in angiosperms.

**Table 3:** Comparison of *ATP/ADP-IPT*s and *tRNA-IPT*s in flowering plants

	*ATP/ADP-IPT*s (non-ancient)	*tRNA-IPT*s (ancient)
Substrate	ADP or ATP	tRNA
Product	iPs and *t*Zs	*c*Zs
Gene structure^a^	Intron-less	Intron-rich
Origin^a^	Last common ancestor of flowering plants	Last common ancestor of eukaryotes
Copy number^a^	2–21 (average 8.13)	2–3 (average 2.33)
Expression pattern^a^	Tissue-specific	Constitutive
Abiotic stress responses^a^	Highly responsive	Generally non-responsive
Mutant phenotypes	Increased lateral root primordia and lateral root numbers^21^, decreased numbers of vascular bundles and cells^22^, reduced shoot apical meristem size, reduced inflorescence^14^, inability to form cambium^22^; higher tolerance to drought or salt stress^23^	Small and chlorotic plants^14^, reduced root growth but similar lateral root number compared with the wild-type^20^
Overexpression phenotypes	Enhanced shoot branching and increased floral numbers along with abnormal floral organ development^39^	Not reported
Effects of exogenous application	Effects of exogenous iP/*t*Z-type cytokinins: promotion of callus formation^22^, shoot initiation^41^, lateral root formation^21^, and the development of vascular cambium, shoot apical meristem, inflorescence stems, and ovules^14,22^	Effects of exogenous *c*Z-type cytokinins: slight promotion of callus formation, shoot regeneration^22,41^, callus growth and the retention of chlorophylls^15,16,42^, but requires much higher concentrations than *t*Zs
Proposed roles in angiosperms	Regulating organ development and abiotic stress responses	Housekeeping: maintaining basic cellular functions

^a^Based on the analyses in this study

### Different origins of *ATP/ADP-IPT*s and *tRNA-IPT*s

According to phylogenetic analyses, Lindner et al.^25^ hypothesized that Class I *tRNA-IPT*s have a mitochondrial origin and that Class II *tRNA-IPT*s are derived from a duplication of a Class I *tRNA-IPT* gene in the plant lineages. Nishii et al.^27^ later proposed that Class II *tRNA-IPT*s originated from eukaryotic genes. However, based on a broad and balanced sampling of species from major lineages of all three domains of life (eukaryotes, bacteria, and archaea), our analyses indicate that the coexistence of Class I and II *tRNA-IPT*s can be traced back to the last common ancestor of eukaryotes. Nonplant eukaryotes, except *D. discoideum*, were found to contain both Class I and Class II *tRNA-IPT* homologs for the first time in this study. The reason that Nishii et al.^27^ did not observe this is likely because of their limited sampling of nonplant eukaryotic species. These nonplant species belong to different supergroups that are considered to have diverged from the base of the eukaryotic tree^32^; thus, our results show that the two classes of *tRNA-IPTs* coexisted in the last eukaryotic common ancestor (LECA).

In this study, we identified two *tRNA-IPT* homologs in archaeal species for the first time. *tRNA-IPT*s have been previously reported to be widely distributed in other kingdoms but not in archaea^19,26,31^. Although it is unclear whether the archaeal *tRNA-IPT*s are functional, their identification reveals that *tRNA-IPT* sequences are distributed throughout all three kingdoms and suggests the possibility of an archaeal origin for *tRNA-IPT*s. Our phylogeny indicates that bacterial or archaeal species contain only one type of *tRNA-IPT* homolog (Fig. 1 and Table S1). The Class I *tRNA-IPT*s are likely to have a closer relationship with the homologs in proteobacteria and proteobacteria-related bacteria, whereas the Class II *tRNA-IPT*s are more closely related to other bacterial groups and the two archaeal species. Eukaryotes are believed to have resulted from the initial endosymbiotic event involving α-proteobacterial and archaeal cells^28^. Therefore, we propose that Class I *tRNA-IPT*s may be derived from the α-proteobacteria that were involved in the initial endosymbiotic event. The Class II *tRNA-IPT* gene in the LECA appears to have two alternative origins; it is derived from either the archaeal ancestor of eukaryotes or a nonproteobacteria ancestral homolog that was introduced into the LECA via horizontal gene transfer. Because *tRNA-IPTs* are present in the latest common ancestor of eukaryotes, these *tRNA-IPT*s are ‘ancient’ CK biosynthesis genes in angiosperms.

Our phylogenetic and motif analyses indicate that *ATP/ADP-IPT* genes are derived from Class II *tRNA-IPT*s. Most *ATP/ADP-IPT* genes contain no or only one intron, whereas Class II *tRNA-IPT*s contain many introns. This difference in gene structure suggests that either the original *ATP/ADP-IPT* gene was generated via a retroposition duplication of a Class II *tRNA*-*IPT* gene or that intron loss occurred soon after its emergence. Intronless daughter gene(s) and sequences of short direct repeats are the two main molecular features of retroposition^33,34^. We searched the ~100 kb regions upstream to downstream of all the *ATP/ADP-IPT* genes in *Arabidopsis* but did not find any featured short direct repeat sequences. Consequently, although Class II *tRNA-IPT*s account for the origin of *ATP/ADP-IPT* genes, the duplication mechanism is still unclear. Nevertheless, because the evidence from previous studies and the current study clearly demonstrates that the *ATP/ADP-IPT* genes emerged in flowering plants, we call them ‘non-ancient’ CK biosynthetic genes relative to the *tRNA-IPT*s, which we call ‘ancient’ CK biosynthetic genes.

In addition, *ATP/ADP-IPT*s were previously proposed to only be present in flowering plants^19^. In our phylogeny of *IPTs* from all three domains of life, four of the five gymnosperm *IPT*s branch with *tRNA-IPTs*, while the rest (*PaIPT3*) phylogenetically clusters with angiosperm *ATP/ADP-IPT*s (Fig. 1). Although the support for this clustering is not great, this close relationship suggests that *PaIPT3* is probably an *ATP/ADP-IPT* gene. *ATP/ADP-IPT*s are responsible for the biosynthesis of *t*Z-type CKs. It has been observed that *t*Zs, rather than *c*Zs, are predominant in vegetative shoots/leaves in most gymnosperms and angiosperms^35^. By comparison, in seedless plants, whose *IPTs* all phylogenetically branch with *tRNA-IPTs*, *c*Zs are the most abundant CKs. The general CK composition of gymnosperms, which is similar to that of angiosperms but distinct from that of seedless plants, suggests that gymnosperms have a similar *IPT* gene composition to that of angiosperms; that is, gymnosperms may already contain *ATP/ADP-IPT* genes. Future investigations on the biochemical characteristics, including preferred substrates and products, of gymnosperm IPT proteins are needed to functionally confirm that *ATP/ADP-IPT* genes originate from gymnosperms.

### *tRNA-IPT*s and *c*Z-type CKs likely play housekeeping roles, while *ATP/ADP-IPTs* and iP/*t*Z-type CKs may be involved in organ development and stress responses

Based on the following lines of evidence, we hypothesize that in angiosperms, *tRNA-IPT*s and associated *c*Z-type CKs mainly play housekeeping roles to maintain basic cellular functions, whereas *ATP/ADP-IPT*s and their products, the iP-type and *t*Z-type CKs, are more likely to be involved in the regulation of organ development and stress responses.

First, expression profiling indicates the functional differences between *ATP/ADP*-*IPTs* and *tRNA-IPT*s. Our genomic transcriptome and qPCR analyses of *F. vesca*, for instance, demonstrated that *ATP/ADP-IPT* genes display drastic changes at the expression level in various tissues/organs and developmental stages (Figs. 4 and 5). In contrast, *tRNA-IPT*s are constitutively and relatively stably expressed in all tissues and developmental stages throughout the plant. Similar expression patterns of the *ATP/ADP-IPT* and *tRNA-IPT* genes have also been observed in the tissues/organs of *Arabidopsis*^36^, *Z. mays*^37^ (summarized in Figs. S3 and S4, respectively) and other species. Therefore, the expression patterns of the *IPT* genes suggest that *ATP/ADP-IPT*s play regulatory roles in organ development in angiosperms, while *tRNA-IPT*s most likely act as housekeeping genes.

Second, the evolutionary histories of *ATP/ADP-IPTs* and *tRNA-IPT*s in angiosperms support their different roles. Our results demonstrate that *tRNA-IPT*s are conservatively retained as two or three copies in flowering plants, while *ATP/ADP-IPT*s have undergone considerable expansions and are highly variable in gene number (from 2 to 21) among species. Moreover, we found that the *ATP/ADP-IPT* genes (*AmIPT2* and *4*) were rarely expressed in the flower buds of *A. trichopoda* (Fig. S2), an extant basal flowering plant with primitive flower tissues^38^. In contrast, most *ATP/ADP-IPT* genes are highly expressed in different floral tissues or developmental stages in core angiosperms, such as *F. vesca*, *Arabidopsis*, and maize. This discrepancy suggests that the expansion and functional diversification of *ATP/ADP-IPT* genes in eudicots and monocots have contributed to their diverse regulatory roles in floral organs.

Third, the manipulation of the expression of different *ATP/ADP-IPT* and *tRNA-IPT* genes gives rise to different phenotypic variations. The *tRNA-atipt9* single and *tRNA-atipt2/9* double mutant plants of *Arabidopsis* exhibited an overall small and chlorotic phenotype with no obvious alterations in organ differentiation or development^14,20^. In contrast, the *ATP/ADP-IPT*-mutant plants *atipt3* or *atipt5* had increased numbers of lateral roots and lateral root primordia^21^. The triple mutant *atipt3;5;7* exhibited a decrease in dedifferentiation ability, which resulted in the reduced formation of callus tissues from root explants after wounding^22^. The quadruple *ATP/ADP-IPT* mutant *atipt1;3;5;7* produced fewer leaves, altered inflorescence^14^, and was unable to develop cambium in the root and stem^22^. Additionally, the overexpression of an *ATP/ADP-IPT* gene resulted in reduced root growth, enhanced shoot differentiation, increased branching, and increased flower number along with abnormal floral organ development^39^. Altogether, the phenotypic changes of these mutant or transgenic plants indicate that *ATP/ADP-IPT*s regulate organ development in angiosperms but that *tRNA-IPT*s do not.

Fourth, the iP/*t*Z-type and *c*Z-type CKs produced by *ATP/ADP-IPT*s and *tRNA-IPT*s, respectively, have been shown to be differentially involved in developmental processes in flowering plants. Higher levels of iPs and *t*Zs than *c*Zs have been detected in apical meristems, fertilized embryos and other tissues with high dedifferentiation and differentiation activities^16,40^. Furthermore, *t*Zs were much more potent than *c*Zs in an assay of callus and shoot differentiation^15,16,41,42^. It has been shown that the endogenous content of *t*Zs was substantially elevated within 12 h after the wounding of root explants, which reflected a similar but earlier increase in comparison with the expression of core cell cycle regulator genes key for callus formation^22^. A decrease in *t*Zs and iPs in the *ATP/ADP-IPT* mutant *atipt3;5;7* resulted in reduced callus formation, while an increase in *t*Zs and iPs by the overexpression of *Agrobacterium IPT* (*AMP-IPT*) dramatically enhanced the production of callus and shoots^43,44^. Additionally, *t*Zs and iPs have been shown to be important in regulating the development of shoot apical meristem, vascular cambium, lateral roots, inflorescence stems, and ovules^14,21,22^. On the other hand, few changes in *c*Z levels were detected during wound-induced callus formation, indicating that endogenous *c*Zs were unlikely to play a significant role. The effects of the overproduction of *c*Zs or prenylated tRNAs remain unknown, as there has been no report on *tRNA-IPT* overexpression so far. However, the exogenous application of *c*Zs are relatively ineffective in stimulating shoot and other organ development under in vitro conditions^15,16,41,42^. *c*Z-deficient *tRNA-ipt* mutant plants of *Arabidopsis* are chlorotic and show much reduced plant sizes^14,20^, suggesting that *c*Z-type CKs may be essential for maintaining the basic functions of cells. Taken together, the results suggest that *ATP/ADP-IPT*s and the associated iP-type and *t*Z-type CKs function as important regulators of angiosperm organ development, whereas *tRNA-IPT*s and the associated *c*Z-type CKs play basic housekeeping roles in angiosperm cells.

Fifth, ATP/ADP-IPTs and tRNA-IPTs catalyze different biochemical reactions that produce two distinct types of CKs, the iPs/*t*Zs- and the *c*Zs, respectively^14^. ATP/ADP-IPTs catalyze the rate-limiting step of the de novo biosynthesis of CKs, directly generating iP and *t*Z nucleotides or nucleosides that are subsequently converted into their active free-base forms^19^. By comparison, the tRNA-IPTs in angiosperms primarily catalyze the isopentenylation of adenine in tRNA to produce *c*Zs from the degradation of prenylated tRNAs^14,19^. Although the function of prenylated tRNA remains unclear in plants, studies in bacteria, yeast, and mammalian cells have shown that tRNA prenylation is important for translational efficiency and fidelity^45^ by preventing frameshifts and the nonsense suppression of the codon UAA^40,46^. Moreover, despite the relatively high expression of *tRNA-IPT*s throughout the plant, the content of endogenous *c*Zs is generally low^16^. The application of *c*Z to *35S:AtCKX7* plants that had reduced *c*Z levels could correct their shorter root phenotype, whereas the application of *c*Z to *tRNA-atipt2/9* plants could not^20^. These results indicate that the reduced root growth observed in *tRNA-atipt2/9* mutants may not be due to the loss of *c*Z production but more likely results from the loss of tRNA prenylation, suggesting that another housekeeping function of *tRNA-IPT*s is to maintain translational accuracy, which is essential for normal cellular activities.

Sixth, similar to their roles in organ development, angiosperm *ATP/ADP-IPT*s have also diversified in response to environmental stresses. Our results show that the expression levels of all three expressed *ATP/ADP-IPT* genes in *F. vesca* are substantially reduced under heat, cold, drought, or salt stress (Fig. 6). Studies in other angiosperms have observed similar findings. In rice, salinity suppressed, whereas cold or drought stress enhanced the expression of most *ATP/ADP-IPT* genes^47^. In *Arabidopsis*, the expression patterns of *ATP/ADP-IPT* genes under stress conditions were also differentially regulated in shoots and roots^47^. Moreover, the *t*Z-type CKs that are produced by ATP/ADP-IPT vary upon environmental stress, depending on the type and intensity of the stress treatment^40^. In contrast, *tRNA-IPT* genes display relatively little variation in their expression level when plants are subjected to environmental stresses (Fig. 6), as shown in previous studies^37,40^. Although the elevation of *c*Z levels has been found under stress conditions, it is generally recognized that *c*Zs are only a byproduct of increased prenylated tRNA turnover but are not produced from the expression of the *tRNA-IPT* genes^40,48^. These observations indicate that *tRNA-IPT*s are more likely to play a housekeeping role, whereas *ATP/ADP-IPT*s make an important contribution to the regulation of plant responses to environmental stresses.

Additionally, along with a reduction in the expression levels of *ATP/ADP-IPT*s under abiotic stress conditions, *ATP/ADP-IPT* mutant plants that contain deficient iP/*t*Z-CK levels but little changed *c*Z levels, such as the quadruple *atipt1;3;5;7 Arabidopsis* mutant, display increased tolerance to drought and salt stress^23^. There have been few experiments that have shown the effects of altered *c*Z content on plant responses to abiotic stress. However, the overexpression of *AtCKX* isoforms, which led to substantial decreases in iP/*t*Z-CKs but either largely reduced or unchanged *c*Z levels, resulted in improved levels of tolerance to drought or salt stress^23^. Therefore, compared with iPs/*t*Zs, variations in *c*Z content appear to have little effect on plant tolerance to abiotic stress. It has been suggested that *c*Zs produced by tRNA-IPTs are more involved in the maintenance of a basal level of CK activity under growth-limiting conditions^16,40^. Accordingly, it is more likely that the elevation of *c*Z levels in stressed conditions is associated with their housekeeping role in plant growth and development, whereas iP/*t*Z-type CKs produced by ATP/ADP-IPTs play an important role in the regulation of plant adaptation to environmental stresses.

In summary, based on our results that are presented in this manuscript and previously published data, we hypothesize that *tRNA-IPT*s (ancient CK biosynthesis genes) and the associated *c*Zs play a housekeeping role, whereas *ATP/ADP-IPT*s (non-ancient CK biosynthetic genes) and the associated iP/*t*Z-type CKs play regulatory roles in organ development and stress responses in angiosperms. An accompanying paper in this issue provides additional evidence to support this hypothesis^49^. It is our hope that this hypothesis will stimulate more interest in elucidating the origins, evolutionary significance, and roles of different types of *IPTs* (*tRNA-IPT*s vs. *ATP/ADP-IPT*s) and the associated different types of CKs (iP/*t*Z vs. *c*Z types) in regulating organ development and responses/adaptation in angiosperms.

## Materials and methods

### Homolog identification

A total of 59 whole-genome-sequenced species sampled from major lineages of all three domains of life (eukaryotes, bacteria, and archaea, Table S1) were first examined for the presence of *IPT* genes. To explore the evolution of *IPT* genes in land plants, 19 sequenced species belonging to every major lineage of land plants (Table S2) were next sampled and analyzed. The complete genomic sequences and corresponding annotation information for all these species were downloaded from the JGI and NCBI databases.

The hidden Markov model profile of the IPPT domain (PF01715) was downloaded from the Pfam database^50^, which was used as a query to search for homologous sequences in the proteome data sets. Sequences with an expected value (*E*-value) < 10^−4^ were considered candidates. The chromosomal locations of all candidates were verified to remove redundant sequences. Short proteins with lengths <100 aa were removed^51^. We further confirmed the presence of the IPPT domain in each candidate using the PFAM (http://pfam.xfam.org/search)^50^ and SMART databases (http://smart.embl-heidelberg.de/)^52^ with an *E*-value cut off <1e^−10^. For archaea, TBLASTN was first used to find potential hits for the IPPT domain under the threshold of 1.0. For the significant hits, the corresponding amino acid sequences of the high-scoring segment pairs (HSPs) were retrieved to assess the presence or absence of the IPPT domain via the PFAM and SMART databases.

### Phylogenetic analyses

The full-length amino acid sequences of all identified IPTs were aligned using ClustalX 2.0^53^. The best-fit model for protein evolution was selected using the Model-Generator program^54^. A phylogenetic tree of the IPT family was constructed via the maximum-likelihood (ML) method using PhyML (version 3.0) software^55^ with the JTT evolutionary model. The tree topology was reconstructed using the best of nearest-neighbor interchange (NNI) and subtree pruning and regraphing (SPR) methods^55^. Branch supports were estimated using an approximate likelihood ratio test with a Shimodaira–Hasegawalike procedure^55^. The phylogenetic trees were visualized in FIGTREE.

### Gene structure, protein motif, and synteny analyses

The relative intron positions in the *IPT* genes were extracted from the annotated whole-genome data sets of 19 land plants. The gene structure was viewed in the online software Evolview (http://www.evolgenius.info/evolview/).

MEME^56^ was used to discover the conserved motifs in IPT proteins from the four representative flowering plants, *A. trichopoda*, *Zea mays*, *A. thaliana*, and *F. vesca*. The parameters were set as follows: the maximum number of motifs, 15; minimum motif width, 6 aa; maximum motif width, 50 aa.

Synteny analyses of the 15 flowering plant genomes were conducted locally using a method similar to that developed for the PGDD (http://chibba.agtec.uga.edu/duplication/)^57^. BLASTP was used to search for potential homologous gene pairs (*E* < 1e^−10^, top five matches) across multiple genomes. These homologous pairs were then used as the input for MCScanX to identify the syntenic regions^29,58^. MCScanX was further used to identify the *IPT* genes resulting from WGD/segmental, tandem, proximal, and dispersed duplications (http://chibba.agtec.uga.edu/duplication/index/downloads).

### Transcriptome data analyses

Transcriptome data from different tissues and developmental stages of *F. vesca*^59,60^ and *A. trichopoda* were downloaded from the SGR (http://bioinformatics.towson.edu/strawberry/) and NCBI (https://www.ncbi.nlm.nih.gov/bioproject/PRJNA212863) databases, respectively. The reads per kilobase per million (RPKM-mapped reads) values were directly retrieved from the website, and the expression levels for the *IPT* genes were plotted on a log_2_ scale.

### Plant growth conditions, stress treatments, and material collection

All plant materials were collected from a seventh-generation inbred line of *F. vesca*, Ruegen (kindly provided by Janet Slovin). For the strawberry fruit collection, strawberry plants were grown in 10 cm × 10 cm pots in a greenhouse with a 12 h photoperiod at 25 °C with 65% relative humidity. Strawberry fruits at the little green (2–4 days after anthesis), big green (8–10 days after anthesis), white, preturning, pink (slight pink flesh and red seeds) and red stages (2–3 days after the pink stage) as well as leaves, immature roots, and mature roots were collected prior to immediate submersion in liquid nitrogen. The tissues from at least three samples were combined to form one biological replicate, and each tissue type utilized three biological replicates.

For the environmental stress experiments, sterile strawberry seedlings were grown in magenta boxes for 2 months in a growth chamber with a 16 h photoperiod at 22 °C and 3000 lx. For the heat shock treatment, the seedlings were transferred to a growth chamber at 38 °C and 3000 lx and were collected at 1, 3, 4 (3 h heat shock and 1 h recovery at 22 °C) and 8 h (3 h heat shock and 5 h recovery at 22 °C). Prior to the salinity stress treatment, the seedlings were transferred to 1/2 MS liquid media and cultivated with gentle agitation (100 rpm). After 12 h, the seedlings were transferred to 1/2 MS liquid media supplemented with 150 mM sodium chloride. For drought treatment, the seedlings were placed on filter paper under dim light at 22 °C with 65% relative humidity. For cold treatment, the seedlings were transferred to a growth chamber set at 4 °C (in the dark). Salinity-stressed, cold-stressed, and dehydration-stressed seedlings were collected at 1, 3, and 8 h after the beginning of the treatment. All collected plant materials were immediately submerged in liquid nitrogen prior to RNA processing.

### Quantitative real-time PCR (qRT-PCR) analysis

A modified CTAB method was used for RNA isolation from all the samples mentioned above. The isolated RNAs were treated with DNase I and used for cDNA synthesis using the Primerscript RT Reagent Kit with gDNA Eraser (Takara). qRT-PCR was performed using SYBR Premix Ex Tag (Takara) with the cDNA as the template. The IDT website (http://sg.idtdna.com/site) was used to design the qRT-PCR primers for the *FveIPT* genes (Table S3). The sequence similarity between *FveIPT3* and *FveIPT4* is >90%; thus, we used a single primer set to investigate the combined expression of both genes. The results were analyzed using the −ΔΔCT method^61^ with *GAPDH*^62^ as the control locus. Three biological and three technical replicates were analyzed.

## Supplementary information

Supplementary Information
