# Supplementary material for: Evolution and roles of cytokinin genes in angiosperms 1: Do ancient IPTs play housekeeping while non-ancient IPTs play regulatory roles?
Source: Hortic Res. 2020 Mar 1;7:28. doi: 10.1038/s41438-019-0211-x (PMC7049300; doi:10.1038/s41438-019-0211-x)
Supplement: Supplementary file 1 — Supplementary Information [file 41438_2019_211_MOESM1_ESM.docx]

**Supplementary Information**

**Article title:** Evolution and roles of cytokinin genes in angiosperms 1: Do ancient *IPTs* play housekeeping while non-ancient *IPTs* play regulatory roles?

**Journal:** Horticulture Research

**Authors:** Xiaojing Wang, Shanshan Lin, Decai Liu, Lijun Gan, Richard McAvoy, Jing Ding, Yi Li

**Corresponding authors:**

**Jing Ding**, State Key Laboratory of Crop Genetics and Germplasm Enhancement and College of Horticulture, Nanjing Agricultural University, Nanjing, P. R. China; e-mail: jding@njau.edu.cn;

**Yi Li**, State Key Laboratory of Crop Genetics and Germplasm Enhancement and College of Horticulture, Nanjing Agricultural University, Nanjing, P. R. China; Department of Plant Science and Landscape Architecture, University of Connecticut, Storrs, CT 06269, USA (Yi Li holds a no pay visiting professor position at Nanjing Agricultural University); e-mail: yi.li@uconn.edu.


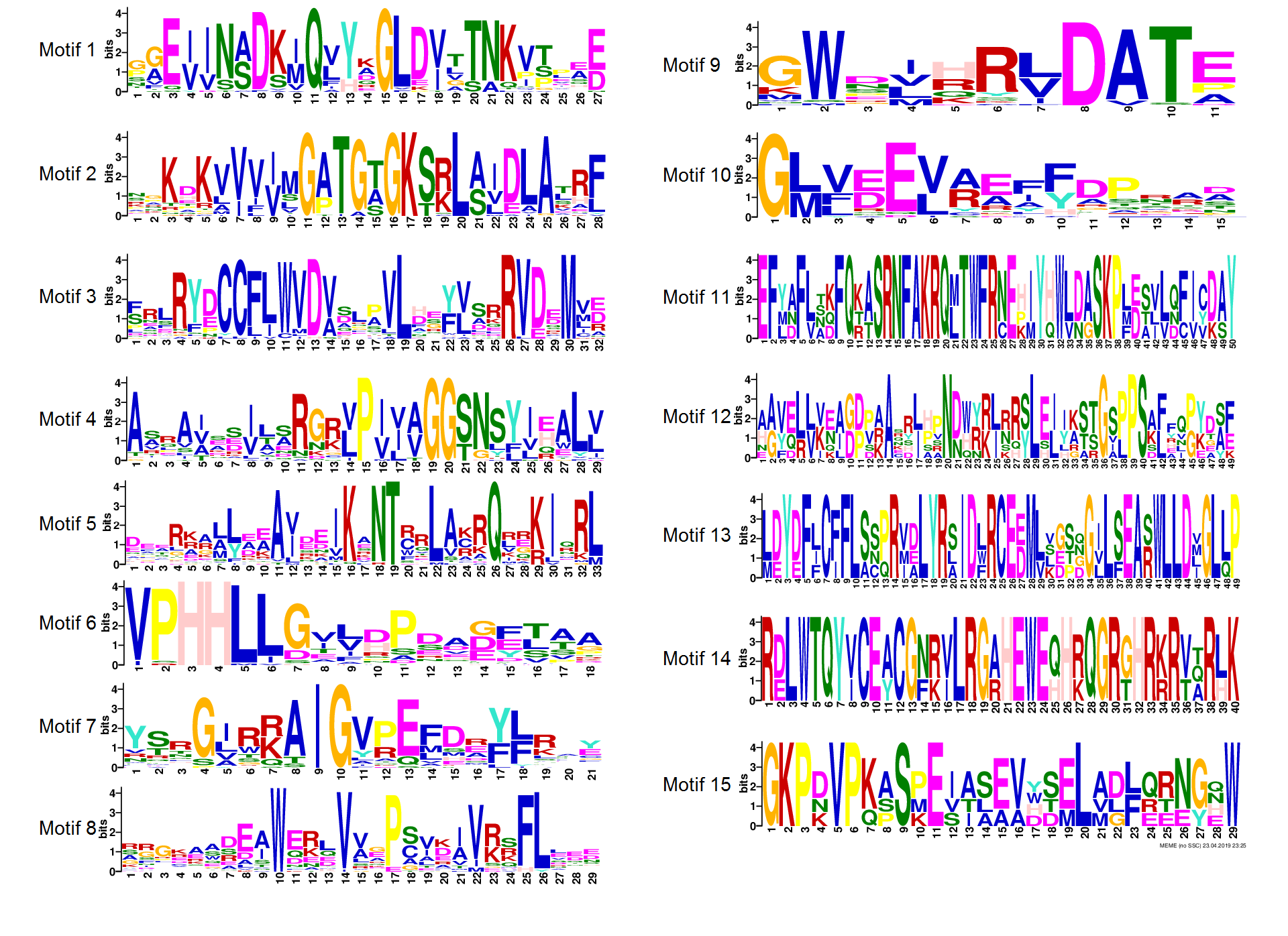


**Fig. S1 Symbols of the motif structures in Fig. 3.**


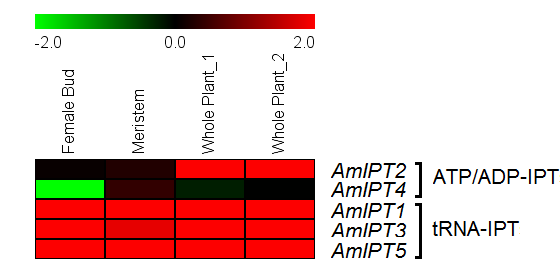


**Fig. S2 Expression pattern of *AmIPT* genes in different tissues from *A. trichopoda.*** The data were based on the transcriptomic results reported by the *Amborella* Genome Project^43^. Expression levels were calculated with the log_2_ scale. Green and red indicate lower and higher transcript abundance, respectively.


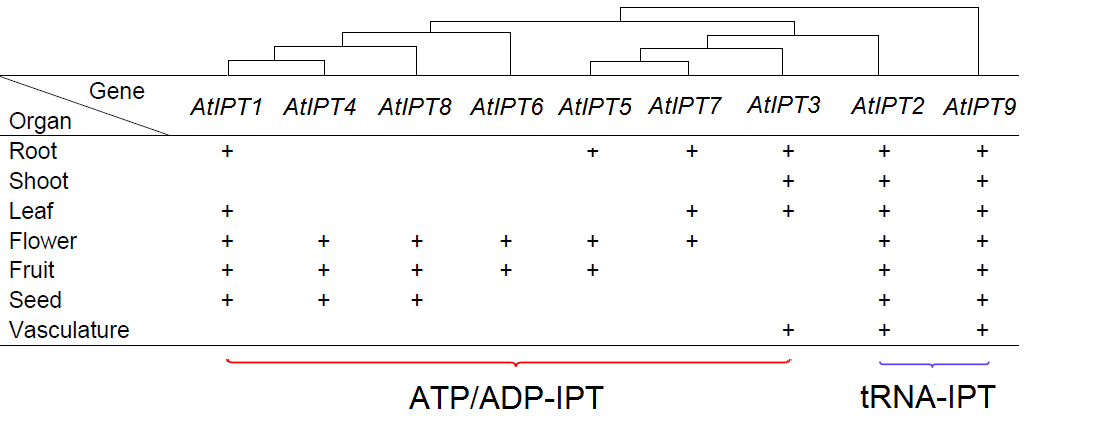


**Fig. S3 Expression pattern of *AtIPT*::*GUS*.** Organ-specific expression patterns are summarized based on the promoter::GUS staining results obtained by Werner et al.^41^. ‘+’ denotes strong GUS activity.


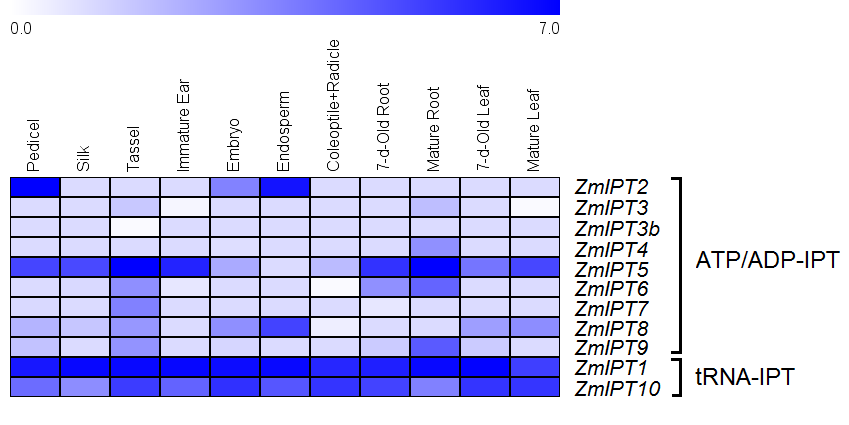


**Fig. S4 Expression patterns of *ZmIPT* genes in different tissues/organs based on qPCR results obtained by Vyroubalova et al.^42^.** The legend shows the expression levels of the *IPT* genes. White and blue indicate lower and higher transcript abundance, respectively, compared to that of the relevant controls.

**Table S1 Numbers of *IPT* homologs (*ATP/ADP-IPT*, *AMP-IPT*, Class I and Class II *tRNA-IPT*) identified in the sampled species**

| Domain | Supergroup | Phylum/group | Abbr.^a^ | Species | Number of IPTs | | | | |
| --- | --- | --- | --- | --- | --- | --- | --- | --- | --- |
|  |  |  |  |  | Total | class I tRNA | class II tRNA | AMP | ATP  /ADP |
| Bacteria |  | Elusimicrobia | Elu | *Elusimicrobia bacterium GWA2_61_42* | 1 | 1 |  |  |  |
|  |  | Chlamydiae | Cca | *Chlamydia caviae* | 1 | 1 |  |  |  |
|  |  | Chlamydiae | Cfe | *Chlamydia felis* | 1 | 1 |  |  |  |
|  |  | Chlamydiae | Cpn | *Chlamydophila pneumoniae CWL029* | 1 | 1 |  |  |  |
|  |  | Rokubacteria | Rok | *Candidatus Rokubacteria bacterium 13_1_40CM_4_69_39* | 1 | 1 |  |  |  |
|  |  | Spirochaetes | Lil | *Leptonema illini DSM 21528* | 1 | 1 |  |  |  |
|  |  | Proteobacteria (Alpha-) | Rhi | *Rhizobium sp. YS-1r* | 1 | 1 |  |  |  |
|  |  | Proteobacteria (Alpha-) | Avi | *Agrobacterium vitis* | 1 | 1 |  |  |  |
|  |  | Proteobacteria (Zeta-) | Mfe | *Mariprofundus ferrooxydans M34* | 1 | 1 |  |  |  |
|  |  | Proteobacteria (Acidithiobacillia) | Tte | *Thermithiobacillus tepidarius DSM 3134* | 1 | 1 |  |  |  |
|  |  | Proteobacteria (Beta-) | Bpr | *Beta proteobacterium AAP51* | 1 | 1 |  |  |  |
|  |  | Proteobacteria (Gamma-) | Xal | *Xanthomonas albilineans* | 2 | 1 |  | 1 |  |
|  |  | Proteobacteria (Gamma-) | Oce | *Oceanimonas sp. GK1* | 1 | 1 |  |  |  |
|  |  | Amesbacteria | - | *Candidatus Amesbacteria bacterium GW2011_GWA2_42_12* | 0 |  |  |  |  |
|  |  | Berkelbacteria | Ber | *Candidatus Berkelbacteria bacterium CG2_30_43_20* | 1 |  | 1 |  |  |
|  |  | Parcubacteria | Par | *Parcubacteria group bacterium GW2011_GWA2_37_10* | 1 |  | 1 |  |  |
|  |  | Cyanobacteria | Nos | *Nostoc sp. PCC 7120* | 2 |  | 1 | 1 |  |
|  |  | Firmicutes | Aac | *Alicyclobacillus acidocaldarius LAA1* | 1 |  | 1 |  |  |
|  |  | Chloroflexi | - | *Anaerolineae bacterium CG2_30_58_95* | 0 |  |  |  |  |
|  |  | Actinobacteria | Rfa | *Rhodococcus fascians* | 2 |  | 1 | 1 |  |
|  |  | Atribacteria | Atr | *Candidatus Atribacteria bacterium CG2_30_33_13* | 1 |  | 1 |  |  |
|  |  | Ignavibacteriae | Ign | *Ignavibacteria bacterium CG1_02_37_35* | 2 |  | 2 |  |  |
| Archaea |  | Bathyarchaeota  (TACK group) | Mcr | *Miscellaneous crenarchaeota group archaeon SMTZ-80* | 1 |  | 1 |  |  |
|  |  | Unclassified  Euryarchaeota | Msb | *Candidate divison MSBL1 archaeon SCGC-AAA382N08* | 1 |  | 1 |  |  |
|  |  | Micrarchaeota | - | *Candidatus Micrarchaeota archaeon RBG_16_36_9* | 0 |  |  |  |  |
|  |  | Unclassified Archaea | - | *Archaeon GW2011_AR21* | 0 |  |  |  |  |
|  |  | Nanohaloarchaeota | - | *Candidatus Haloredivivus sp. G17* | 0 |  |  |  |  |
|  |  | Altiarchaeales | - | *Candidatus Altiarchaeales archaeon IMC4* | 0 |  |  |  |  |
|  |  | Theionarchaea | - | *Theionarchaea archaeon DG-70* | 0 |  |  |  |  |
|  |  | Methanobacteria | - | *Methanobacterium paludis strain SWAN1* | 0 |  |  |  |  |
|  |  | Crenarchaeota  (TACK group) | - | *Thermogladius cellulolyticus 1633* | 0 |  |  |  |  |
|  |  | Lokiarchaeaota | - | *Lokiarchaeum sp. GC14_75* | 0 |  |  |  |  |
| Eukaryota | SAR | Cercozoa | Pbr | *Plasmodiophora brassicae* | 2 | 1 | 1 |  |  |
|  |  | Foraminifera | Rfi | *Reticulomyxa filosa* | 1 |  | 1 |  |  |
|  |  | Bacillariophyta | Tps | *Thalassiosira pseudonana CCMP1335* | 1 | 1 |  |  |  |
|  | Excavata | Euglenozoa | Tcr | *Trypanosoma cruzi Dm28c* | 1 |  | 1 |  |  |
|  |  | Fornicata | - | *Spironucleus salmonicida* | 0 |  |  |  |  |
|  | Amoebozoa | Mycetozoa | Ddi | *Dictyostelium discoideum AX4* | 3 | 1 | 1 | 1 |  |
|  |  | Discosea | Aca | *Acanthamoeba castellanii str. Neff* | 2 | 1 | 1 |  |  |
|  | Opisthokonta | Fungi | Sce | *Saccharomyces cerevisiae S288C* | 1 |  | 1 |  |  |
|  |  | Ichthyosporea | Sar | *Sphaeroforma arctica JP610* | 2 | 1 |  | 1 |  |
|  |  | Holozoa | Sro | *Salpingoeca rosetta* | 2 | 1 | 1 |  |  |
|  |  | Metazoa | Hsa | *Homo sapiens* | 1 |  | 1 |  |  |
|  | Incertae sedis | Haptophyta | Chr | *Chrysochromulina sp. CCMP291* | 1 | 1 |  |  |  |
|  |  | Cryptophyta | Cme | *Chroomonas mesostigmatica* | 1 | 1 |  |  |  |
|  |  | Apusozoa | Ttr | *Thecamonas trahens* | 2 | 1 | 1 |  |  |
|  | Archaeplastida | Rhodophyta | Cya | *Cyanidioschyzon merolae strain 10D* | 1 | 1 |  |  |  |
|  |  | Chlorophyta | Cre | *Chlamydomonas reinhardtii* | 1 | 1 |  |  |  |
|  |  | Chlorophyta | Vca | *Volvox carteri* | 1 | 1 |  |  |  |
|  |  | Chlorophyta | Olu | *Ostreococcus lucimarinus* | 2 | 1 | 1 |  |  |
|  |  | Chlorophyta | Ota | *Ostreococcus tauri* | 2 | 1 | 1 |  |  |
|  |  | Charophyta | Kni | *Klebsormidium nitens* | 1 | 1 |  |  |  |
|  |  | Marchantiophyta | Mp | *Marchantia polymorpha* | 2 | 2 |  |  |  |
|  |  | Bryophyta | Pp | *Physcomitrella patens* | 6 | 6 |  |  |  |
|  |  | Lycopodiopsida | Sm | *Selaginella moellendorffii* | 1 | 1 |  |  |  |
|  |  | Spermatophyta | Pa | *Picea abies* | 5 | 1 | 3 |  | 1 |
|  |  | Spermatophyta | Am | *Amborella trichopoda* | 5 | 2 | 1 |  | 2 |
|  |  | Spermatophyta | Zm | *Zea mays* | 11 | 1 | 1 |  | 9 |
|  |  | Spermatophyta | At | *Arabidopsis thaliana* | 9 | 1 | 1 |  | 7 |

^a^Abbreviation of the species name in the gene ID in Fig. 1.

**Table S2 Numbers of *IPT* genes in different groups of the phylogeny shown in Fig. 2a**

| Species | ATP/ADP-IPT | | | | | SUM | tRNA-IPT | | SUM |
| --- | --- | --- | --- | --- | --- | --- | --- | --- | --- |
|  | **I** | | | | **II** |  | **tRNA-I** | **tRNA-II** |  |
|  | **I_a_** | **I_b_** | **I_c_** | **I_d_** |  |  |  |  |  |
| *Arabidopsis thaliana* | 0 | 2 | 1 | 0 | 4 | 7 | 1 | 1 | 2 |
| *Brassica rapa* | 0 | 4 | 2 | 0 | 4 | 10 | 2 | 1 | 3 |
| *[Citrus clementina](http://www.ncbi.nlm.nih.gov/Taxonomy/Browser/wwwtax.cgi?mode=Info&id=2711)* | 2 | 1 | 1 | 0 | 1 | 5 | 1 | 1 | 2 |
| *Solanum lycopersicum* | 9 | 0 | 2 | 0 | 2 | 13 | 1 | 1 | 2 |
| *Medicago truncatula* | 16 | 2 | 1 | 0 | 1 | 20(+1)^a^ | 1 | 1 | 2 |
| *Glycine max* | 2 | 3 | 4 | 0 | 2 | 11 | 1 | 2 | 3 |
| *Fragaria vesca* | 0 | 1 | 1 | 0 | 3 | 5 | 1 | 1 | 2 |
| *Prunus persica* | 1 | 1 | 1 | 0 | 1 | 4 | 1 | 2 | 3 |
| *Malus x domestica* | 2 | 2 | 2 | 0 | 3 | 9 | 2 | 1 | 3 |
| *Populus trichocarpa* | 2 | 2 | 1 | 0 | 2 | 7 | 1 | 1 | 2 |
| *Vitis vinifera* | 1 | 1 | 2 | 0 | 1 | 5 | 1 | 1 | 2 |
| *Nelumbo nucifera* | 0 | 3 | 0 | 0 | 2 | 5 | 1 | 2 | 3 |
| *Zea mays* | 0 | 0 | 0 | 6 | 3 | 9 | 1 | 1 | 2 |
| *Oryza sativa* | 0 | 0 | 0 | 5 | 3 | 8 | 1 | 1 | 2 |
| *Amborella trichopoda* | 1 | | | | 1 | 2 | 2 | 1 | 3 |
| *Pinus taeda* | 0 | | | | 0 | 0 | 0 | 2 | 2 |
| *Picea abies* | 0 | | | | 0 | 0 | 1 | 4 | 5 |
| *Selaginella moellendorffii* | 0 | | | | 0 | 0 | 1 | 0 | 1 |
| *Physcomitrella patens* | 0 | | | | 0 | 0 | 6 | 0 | 6 |

^a^A *Medicago truncatula* ATP/ADP-IPT protein phylogenetically clusters with the *Amborella trichopoda* protein in the ATP/ADP-I group, which could not be classified into the ATP/ADP-II group or any subgroup of the ATP/ADP-I group.

**Table S3 Primers used in this study**

| **Gene name** | **Forward primer** | **Reverse primer** |
| --- | --- | --- |
| *FveGADPH* | CATTCATCACCACCGACTACA | GAAGGGTCTTCTCATCCTTGAC |
| *FveIPT1* | GTGGTTCTGGCCGAGTATTTAT | CACTCCGATAGCCTTTCTCAAC |
| *FveIPT2* | CCTGTGGTGACAAGATCCTTAG | GTGCTGCTCGACAGACAAATA |
| *FveIPT3/4* | ATGCAGCTCTACAAGGGCCTCGAC | GCGCCGCGAGACGGCGGAACTCGG |
| *FveIPT5* | CTCTGGGTGGATGTATCCTTATC | CAAAGAACTCTCTCACCTCCTC |
| *FveIPT6* | GACTCGAACTTCACTGCTACTG | TCCACCAGTGCCTCTATGTA |
| *FveIPT7* | CTGGTATCGTCAGGTCTTTGAC | AGAGTTCACAGGGTTGCTAATC |
